# Prevalence of HIV in slums area: a systematic review and meta-analysis

**DOI:** 10.1186/s12879-023-08877-7

**Published:** 2024-01-05

**Authors:** Meysam Behzadifar, Seyed Jafar Ehsanzadeh, Banafshe Darvishi Teli, Samad Azari, Ahad Bakhtiari, Masoud Behzadifar

**Affiliations:** 1 Social Determinants of Health Research Center, Lorestan University of Medical Sciences, Khorramabad, Iran; 2 English Language Department, School of Health Management and Information Sciences, Iran University of Medical Sciences, Tehran, Iran; 3 Department of Health Economics, School of Health Management and Information Sciences, Iran University of Medical Sciences, Tehran, Iran; 4 Hospital Management Research Center, Health Management Research Institute, Iran University of Medical Sciences, Tehran, Iran; 5 Health Equity Research Center (HERC), Tehran University of Medical Sciences (TUMS), Tehran, Iran

**Keywords:** HIV, Slums, Prevalence, Systematic review, Meta-analysis, Health policy

## Abstract

**Background:**

Human Immunodeficiency Virus (HIV) remains a significant global health burden, particularly affecting vulnerable populations residing in slum areas which is characterized by overcrowding, poverty, and limited access to healthcare services, create an environment conducive to the transmission and spread of HIV. Despite the recognition of this issue, there is a lack of comprehensive understanding regarding the prevalence of HIV in slums. The aim of this study was to systematically synthesize the existing global evidence on HIV prevalence in slum populations.

**Methods:**

A rigorous systematic literature review was conducted by searching multiple electronic databases, including Medline via PubMed, Scopus, Embase, Web of Sciences, and Directory of Open Access Journals (DOAJ), covering the period from January 1, 1990, to March 31, 2023. The quality and risk of bias for each included study were assessed using the Newcastle–Ottawa Scale. The pooled prevalence with its corresponding 95% confidence interval (CI) was calculated using a random-effects model with the Freeman-Tukey double arcsine transformation. The degree of heterogeneity among the studies was evaluated using the I^2^ test. Publication bias was also assessed using Egger's test. Additionally, subgroup analysis was performed to explore potential factors contributing to the observed heterogeneity.

**Results:**

A systematic examination of the relevant literature resulted in the inclusion of a total of 22 studies for the purpose of this meta-analysis. These studies collectively assessed a sizable cohort consisting of 52,802 participants. Utilizing a random-effects model, an estimation of the overall prevalence of HIV in the slum area was determined to be 10% (95% CI: 7–13%). Further delineation through subgroup analysis based on the gender revealed a higher prevalence of HIV among women, standing at 13% (95% CI: 8–19%, 18 studies: I^2^ = 98%), as opposed to men, where the prevalence was found to be 8% (95% CI: 6–12%, 16 studies: I^2^ = 95%). A geographical breakdown of the included studies revealed that Africa exhibited the highest prevalence, with a figure of 11% (95% CI: 9–13%, 18 studies: I^2^ = 98%). Subsequently, studies conducted in the American continent reported a prevalence of 9% (95% CI: 7–11%, 2 studies: I^2^ = 57%). The Asian continent, on the other hand, displayed the lowest prevalence of 1% (95% CI: 0–3%, 2 studies: I^2^ = 94%). Notably, studies employing rapid tests indicated a prevalence of 13% (95% CI: 9–17%, 6 studies: I^2^ = 94%), while those relying on self-reported data reported a lower prevalence of 8% (95% CI: 5–11%, 6 studies: I^2^ = 99%). Moreover, studies utilizing ELISA reported a prevalence of 9% (95% CI: 6–12%, 10 studies: I^2^ = 96%). Finally, it was determined that studies conducted in upper-middle-income countries reported a higher prevalence of 20% (95% CI: 16–24%, 5 studies: I^2^ = 45%), whereas studies conducted in lower- and middle-income countries reported a prevalence of 8% (95% CI: 6–10%, 12 studies: I^2^ = 98%).

**Conclusion:**

The current study elucidates the troublingly high prevalence of HIV infection within slums area. Also, this finding underscores the urgent necessity for targeted and tailored interventions specifically aimed at curtailing the spread of HIV within slums. Policymakers must take cognizance of these results and devote their efforts towards the implementation of effective strategies to mitigate gender disparities, address poverty alleviation, and empower the inhabitants of these marginalized areas.

**Supplementary Information:**

The online version contains supplementary material available at 10.1186/s12879-023-08877-7.

## Background

Slums area are characterized by substandard housing conditions, limited access to clean water and sanitation facilities, and exposure to environmental hazards [1]. These conditions contribute to a multitude of health problems, encompassing infectious diseases like tuberculosis, Human Immunodeficiency Virus (HIV), malaria, and acute diarrheal diseases including cholera, as well as chronic conditions such as respiratory and cardiovascular disease, and malnutrition [2]. The prevalence of these health issues is amplified by the challenging living conditions prevalent in slums, often resulting in inadequate access to healthcare services. Consequently, delays in diagnosis and treatment, as well as poor health outcomes, ensue [3, 4]. Additionally, socio-economic barriers, including the cost of medical care and lack of health insurance or formal identification documents, impede slum residents from seeking essential healthcare [5]. HIV constitutes a critical global health concern, affecting individuals across all age groups, sexes, genders, and socio-economic backgrounds [6]. Those residing in slums face limited access to fundamental amenities such as clean water, sanitation facilities, and healthcare services, thereby elevating the risk of HIV transmission [7]. Moreover, social and economic factors, namely poverty, stigma, discrimination, and reduced education and awareness surrounding HIV, further contribute to the spread of the virus [8]. The challenges faced by slum residents in accessing HIV prevention and treatment services, including condoms, pre-exposure prophylaxis (PrEP), antiretroviral therapy (ART), and HIV testing and counseling, exacerbate the risks of transmission and adversely impact the health outcomes of individuals living with HIV [9]. The socio-economic ramifications of HIV within slums are profound, as the disease escalates healthcare expenses, compromises productivity, and diminishes overall quality of life, thereby perpetuating cycles of poverty and inequality [10]. Several studies have been conducted to estimate the HIV prevalence in slum areas in different countries. However, the reported prevalence rates vary widely across studies, and a systematic review of these studies with a meta-analytical approach is needed to obtain a more accurate estimate of HIV prevalence in slums area.

This study aimed to conduct a comprehensive systematic review and meta-analysis of the available academic literature pertaining to HIV prevalence in slums worldwide. The primary objective was to synthesize data from a diverse range of studies in order to derive a robust and representative estimate of the HIV rate in slums. The implications of this study are multifaceted, particularly in the realm of policymaking and decision-making. Firstly, the provision of up-to-date and accurate estimates of HIV prevalence in slums can significantly inform the development and implementation of effective public health policies and interventions, thereby addressing the burden of HIV. Secondly, the identification of factors associated with a heightened prevalence of HIV in slums would facilitate the formulation of targeted prevention and control strategies, thus contributing to efficient resource allocation. Lastly, this study serves to illuminate key gaps in current knowledge concerning HIV prevalence in slums, consequently pinpointing areas necessitating further research to enhance our comprehension of HIV transmission dynamics and associated risk factors within these settings.

## Methods

Methods This systematic review and meta-analysis were conducted in accordance with the Preferred Reporting Items for Systematic Review and Meta-analyses (PRISMA) guidelines (Appendix 1) [11] and were pre-registered within PROSPERO (CRD42023422791) [12].

### Search strategy

A systematic literature search was performed using multiple electronic databases, namely Medline via PubMed, Scopus, Embase, Web of Sciences, and Directory of Open Access Journals (DOAJ), covering the period from January 1, 1990, to March 31, 2023. The reference search was conducted by two authors under the supervision of a librarian. The search employed specific keywords (prevalence, HIV, and slums) to ensure the inclusion of relevant studies. The search strategy used was as follows: (Prevalence OR seroprevalence OR frequency OR rate OR epidemiology) AND (AIDS OR HIV OR human immunodeficiency viruses) AND (Slum OR informal settlements OR urban refuges OR slum dwellers). The resources obtained from the database search were managed using EndNote Version 20 software. (See search strategy in databases in appendix 2).

In case of conflicting results between the two authors, they sought the guidance of a senior researcher to reach a resolution. If the discrepancy persisted, a third author resolved the disparities through consensus. Moreover, the reference lists of the included studies were thoroughly examined to identify any potential studies relevant to the research topic.

### Inclusion criteria

#### Study design

This research encompasses primary studies employing diverse study designs, such as cross-sectional surveys and intervention-based investigations.

#### Study area

The study focuses on investigations conducted in slum or informal settlement areas across various geographical locations.

#### Language

The review includes studies published solely in the English language due to practical and resource constraints.

#### Publication status

The analysis takes into account both published and unpublished studies, encompassing peer-reviewed journal articles.

#### Publication period

The review encompasses studies published from the beginning of relevant databases up to a specific end date, with the aim of capturing the most recent evidence.

#### Population

This study encompasses individuals residing in slum areas, irrespective of their age, gender, or other demographic characteristics.

#### Outcomes

The analysis includes studies that report the prevalence of HIV among the population residing in slum areas, provided that sufficient data are available to calculate the prevalence. Specifically, studies that incorporate information regarding prevalence, the number of participants, and the number of individuals with HIV in their text are considered.

### Exclusion criteria

The exclusion criteria for this study encompassed the following factors: duplicated results, studies lacking sufficient data to calculate prevalence, and studies where the full text was not accessible. Furthermore, this study excluded letters to the editor, case reports, case series, review studies, conference abstracts, and clinical trials. In the context of this research, both male and female genders were included, while other trends were excluded.

### Risk of bias of individual studies

To evaluate the risk of bias in each individual study, we employed the widely recognized and validated Newcastle–Ottawa Scale (NOS) [13]. In the event of disagreements among the authors, a consensus was reached through discussion. The NOS is an established tool extensively employed for assessing the quality and risk of bias in non-randomized studies included in systematic reviews and meta-analyses. When discrepancies arose regarding the evaluation of studies based on specific criteria, a third party arbitrator facilitated the resolution. Based on the NOS scores, we categorized the studies as follows: a score ranging from 1 to 3 indicating high risk, a score from 4 to 6 denoting moderate risk, and a score from 7 to 9 representing low risk.

### Data extraction

Two authors conducted a thorough analysis of the titles of the studies and independently assessed the full texts to identify studies that met the inclusion criteria. The extracted relevant information includes details such as the first author's name, year of publication, country of origin, number of participants, gender distribution, age range, number of individuals with HIV, diagnostic test employed for HIV diagnosis, and reported prevalence. In situations where the authors encountered conflicting outcomes, they sought guidance from a senior researcher to address any discrepancies. If an agreement could not be reached, a third reviewer stepped in to resolve the discrepancies through consensus. The collection of study data was carried out using a carefully designed form, which underwent approval by the authors' team. In cases where the two authors disagreed on certain aspects of information to be included in the final analysis, the involvement of a third party was necessary to settle the disagreement.

### Statistical analysis

The prevalence of HIV in slums was estimated using the inverse variance method, a commonly employed approach in epidemiological studies. To account for potential variability between studies, the DerSimonian-Laird method was utilized to estimate the between-study variance [14]. Furthermore, 95% confidence intervals (CI) were calculated using a random effects model with the Freeman-Tukey double arcsine transformation, which takes into consideration the heterogeneity among studies [15]. The Freeman–Tukey double-arcsine transformation is a widely used technique for synthesizing prevalence studies in meta-analysis [16]. To evaluate the extent of heterogeneity among the studies, the I^2^ test was employed [17]. Additionally, a Baujat plot was generated to visually explore heterogeneity and identify potential sources of variation [18]. As part of a comprehensive analysis, a subgroup analysis was conducted to investigate the contribution of various factors to the observed heterogeneity [19]. Specifically, factors such as gender, year of publication, sample size, diagnostic test, and NOS scale score were examined [20]. To ascertain the robustness of the findings, a sensitivity analysis was undertaken to gauge the impact of individual studies on the overall results [21]. Furthermore, a meta-regression analysis was performed, incorporating variables such as year of publication, sample size, and NOS scale score to explore their potential influence on the outcomes [22]. To evaluate the presence of publication bias, the Egger's test was employed, providing insights into potential selective reporting of studies [23]. The odds ratios (OR) for gender and place of residence were calculated, shedding light on the associations between these variables and HIV prevalence [24]. All statistical analyses were conducted using R Version 4.2.3 via the *meta* package, a widely used tool in meta-analyses and evidence synthesis. To establish statistical significance, two-sided *P* values were employed, with values less than 0.05 considered statistically significant.

## Results

**Fig. 1:**
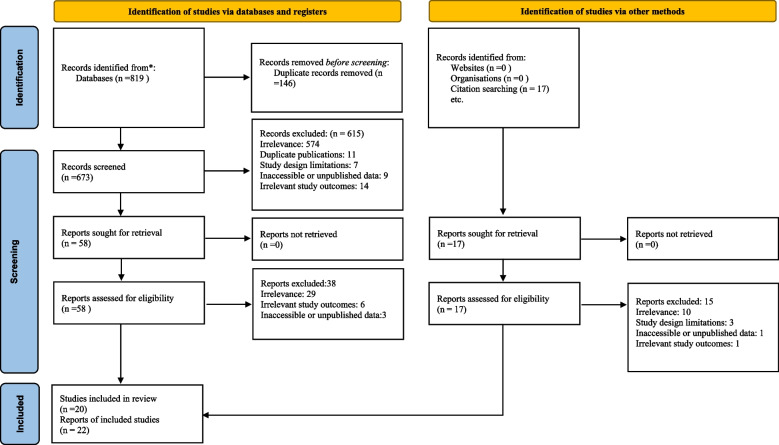
The PRISMA flow diagram

A total of 836 records were identified through an extensive search. Following the elimination of duplicate and irrelevant records, 22 studies were finally included for this systematic review and meta-analysis [25–46]. The process of literature search and study selection is depicted in Fig. 1.

Table 1 presents the characteristics of the selected studies. Out of the included studies, 18 (82%) were conducted in Africa [25, 28–30, 32–39, 41–46], 2 (9%) in North America [26, 40], and 2 (9%) in Asia [27, 31]. Altogether, these studies encompassed a total of 52,802 participants. Where gender-specific data were available, the evaluation included 8,583 males and 12,906 females, with other studies reporting data collectively for both genders.

**Table 1:** The characteristics of included studies

First Author	Year	Country	Test	Age	Sample size
Barongo LR	1992	Tanzania	ELISA	NA	958
Behets FM	1995	Haiti	Rapid test kits	25.9	663
Sabin KM	2003	Bangladesh	ELISA	28	1533
Connolly C	2004	South Africa	ELISA	NA	2753
Ziraba AK	2010	Kenya	Self report	NA	4767
Madise NJ	2012	Kenya	Rapid test kits	15–54	3048
Shrivastava SR	2012	India	ELISA	NA	188
Dalal W	2013	Kenya	Rapid test kits	NA	19,966
Kimani JK	2013	Kenya	Rapid test kits	NA	2409
Cassels S	2014	Ghana	ELISA	18–49	484
Steenkamp L	2014	South Africa	ELISA	18–49	752
Kerubo G	2015	Kenya	ELISA	29.3 ± 9.3	1308
Bouscaillou J	2016	Côte d'Ivoire	ELISA	33.5 ± 8.6	450
Swahn MH	2016	Uganda	Self report	NA	590
Basera TJ	2016	South Africa	Self report	NA	3953
Ssensamba J	2019	Uganda	ELISA	10.8 ± 3	214
Rivera VR	2019	Haiti	Self report	NA	5472
Swahn MH	2019	Uganda	Self report	NA	1103
Baluku JB	2020	Uganda	Rapid test kits	NA	272
Gibbs A	2020	South Africa	ELISA	NA	543
Culbreth R	2020	Uganda	Self report	17 ± 1.3	1134
Bolarinwa OA	2022	South Africa	Rapid test kits	NA	242

**Fig. 2:**
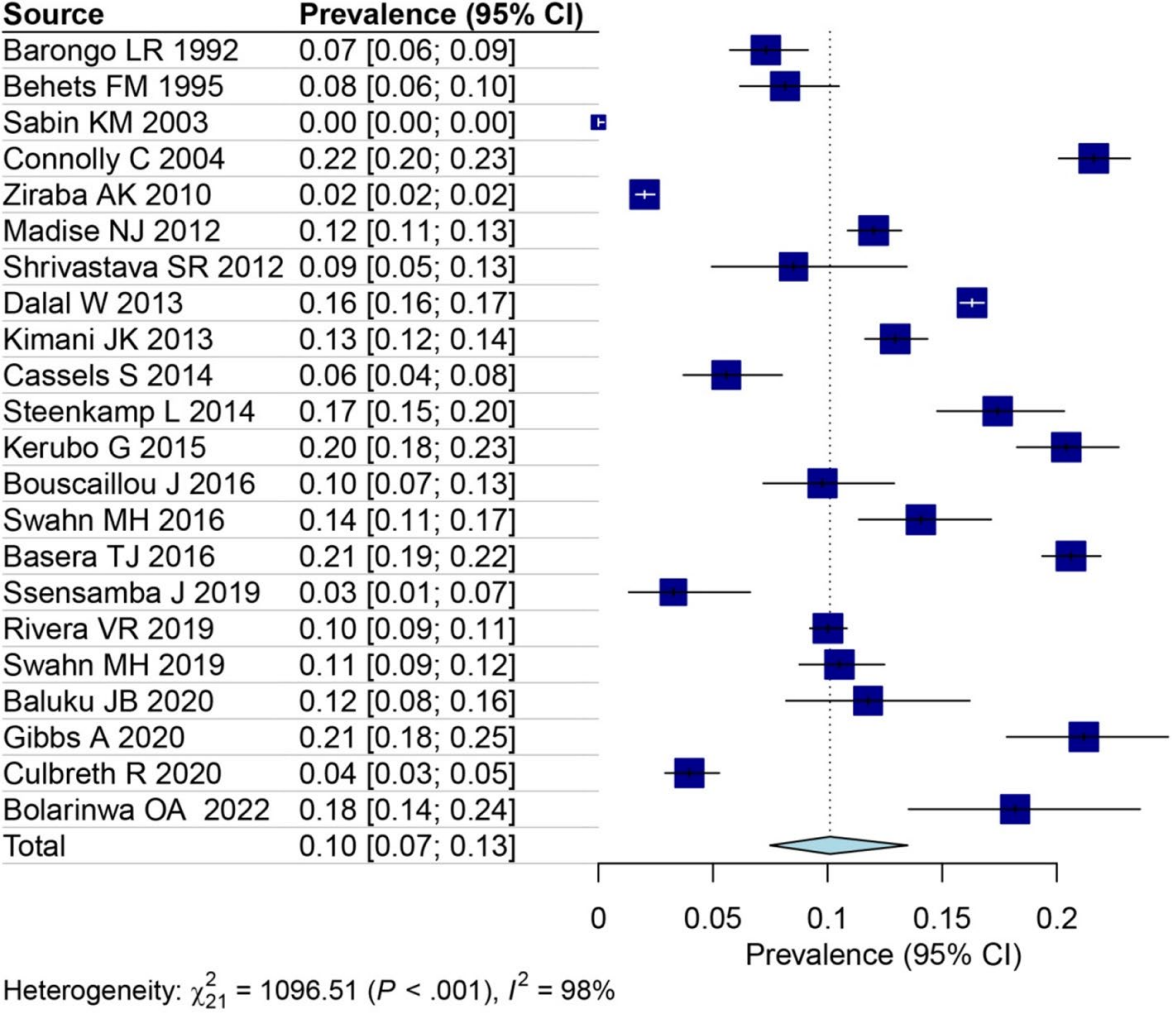
The overall HIV prevalence in slums area

Risk of bias assessment scores based on the NOS for the selected studies are provided in Appendix 3. Among the included studies, the majority (68.1%) were classified as having a low risk of bias [25–28, 30–33, 36–38, 41–44], while the remaining 7 studies (31.9%) were deemed to have a moderate risk of bias [29, 34, 35, 39, 40, 45, 46]. Utilizing a random-effects model, the overall prevalence of HIV in slums area was estimated to be 10% [95% CI: 7–13%, tau^2 = 0.57]. This result demonstrates a high level of heterogeneity, as was observed at I^2^ = 98% (Fig. 2).

Sensitivity and cumulative analyses were conducted to assess the stability of the results. The sensitivity analysis revealed that the findings were robust and reliable (Appendix 4 for additional details).

**Fig. 3:**
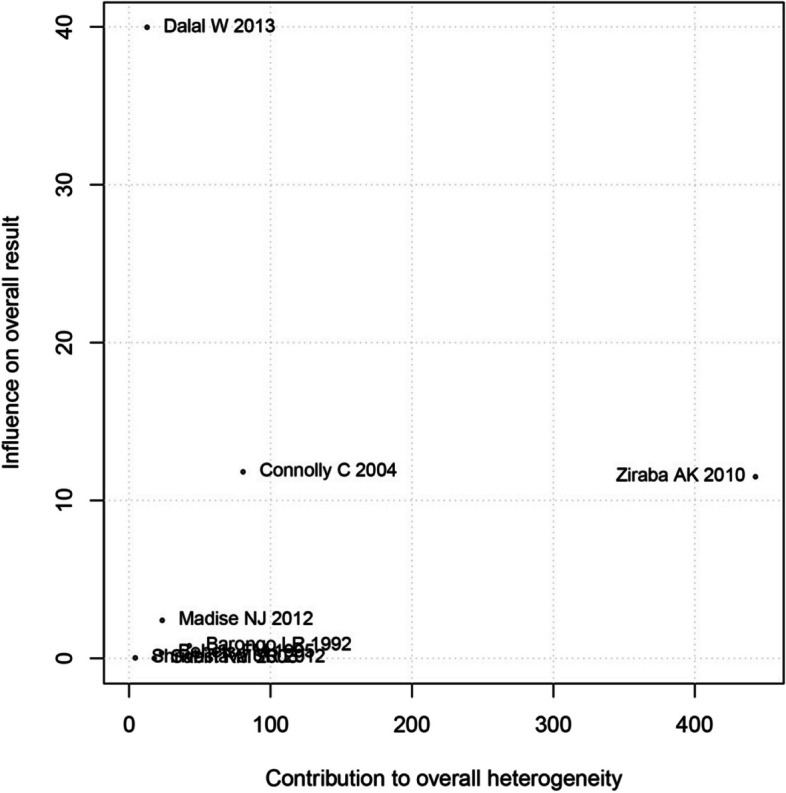
The Baujat plot

Given the high heterogeneity observed among the studies, a Baujat plot (Fig. 3) was constructed to visually represent the variations in prevalence. The analysis showcased that the majority of studies exhibited similar prevalence rates, with the exception of Connolly C et al. [28], Ziraba AK et al. [29], and Dalal W et al. [32], which reported notably higher proportions. In order to obtain a more accurate estimate, these three studies were excluded from the analysis. Consequently, the prevalence of the condition was determined to be 10% (95% CI: 8–14%; I^2^ = 97%, tau^2 = 0.37).

**Fig. 4:**
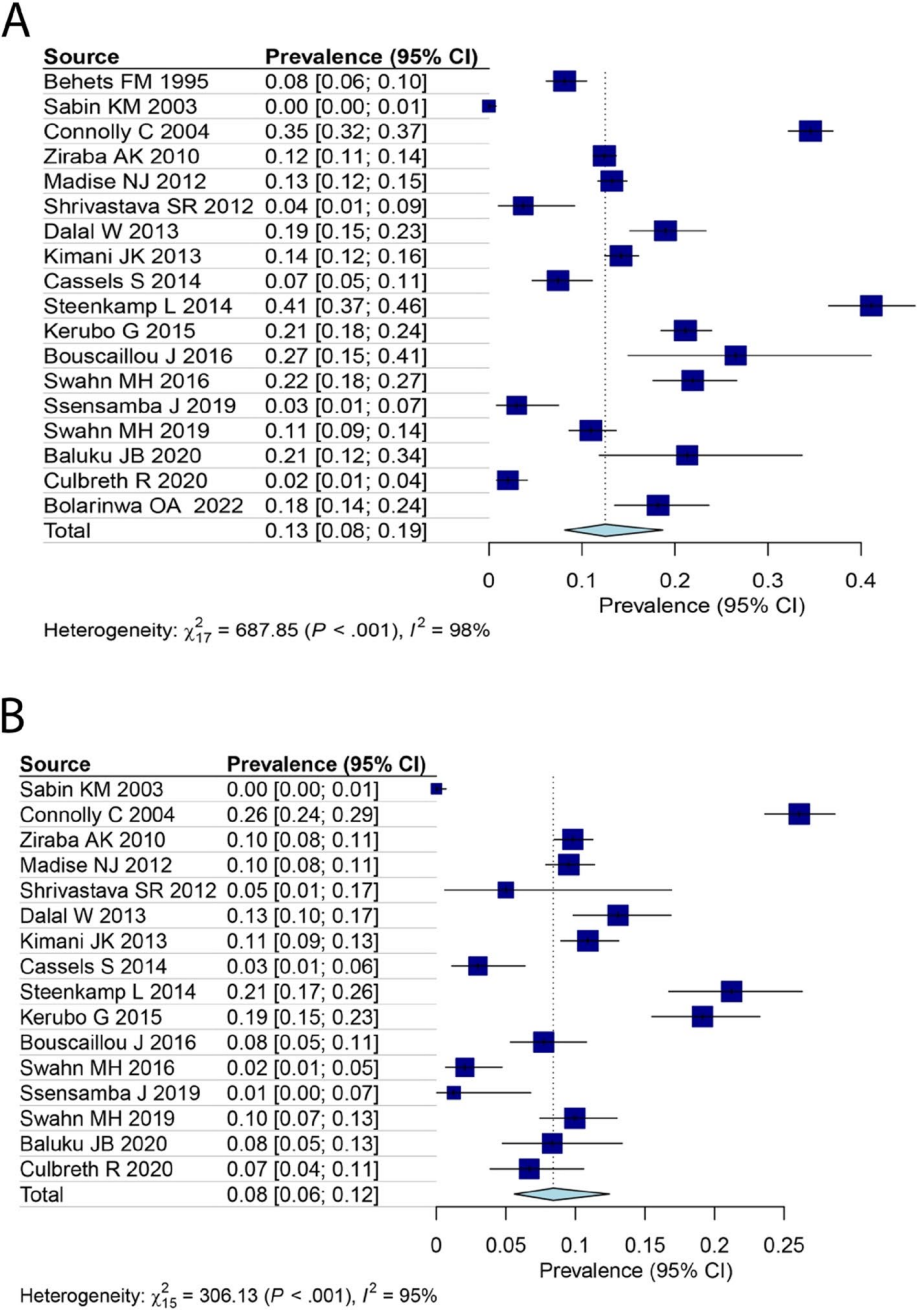
The prevalence of HIV among female (**A**) and male (**B**) in slums area

**Fig. 5:**
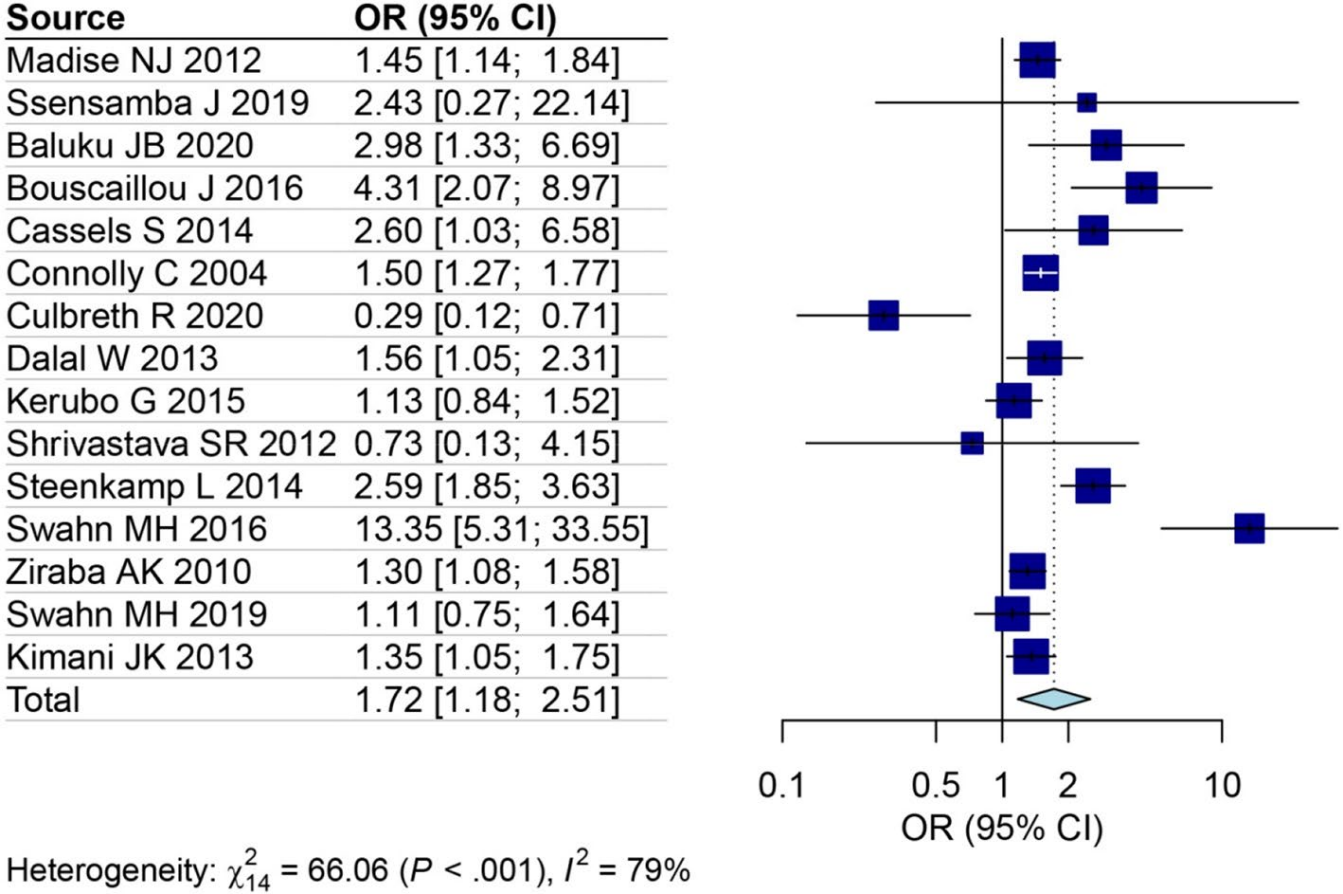
The odds ratio of HIV among females living in slums area compared to males

**Fig. 6:**
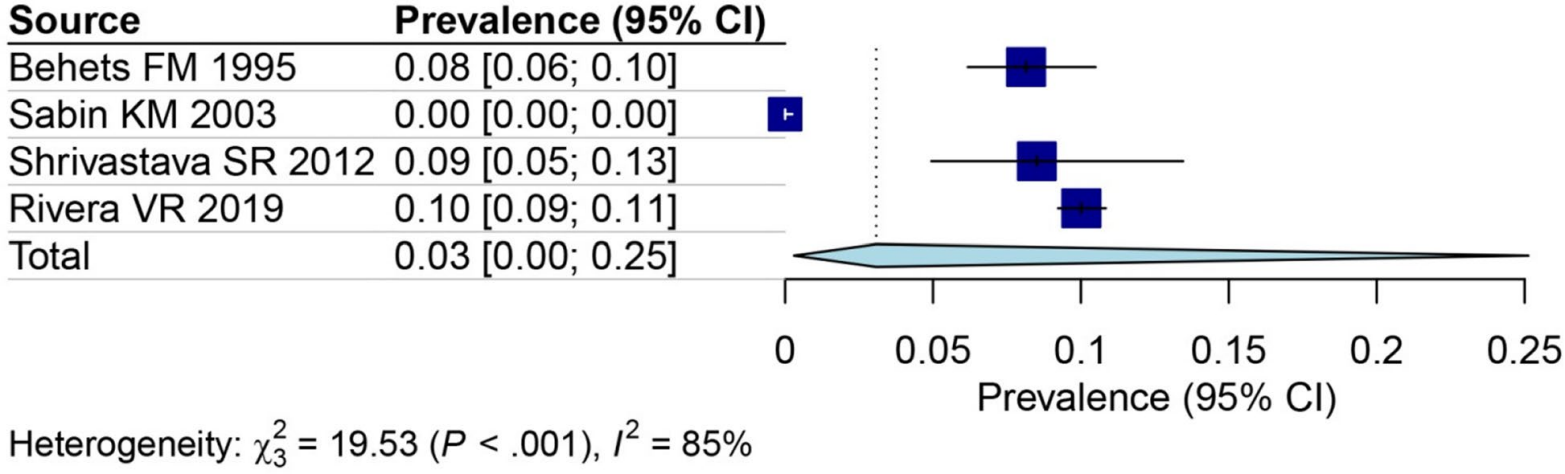
The HIV prevalence in slums area in the four non-African studies

This study aimed to investigate gender-based variations in the prevalence of HIV in slum populations. By analyzing data from 18 studies focused on women [26–36, 38, 39, 41–44, 46] and 16 studies centering on men [27–36, 38, 39, 41–44], the prevalence of HIV was calculated for each gender group. The results indicated that the prevalence of HIV among women (13%, 95% CI: 8–19%) was higher than that of men (8%, 95% CI: 6–12%) (Fig. 4A for females and Fig. 4B for males). Furthermore, based on data from 15 studies that included both genders [28–36, 38, 39, 41–44], an OR was calculated to evaluate the risk of HIV in females living in slums compared to males. The OR was determined to be 1.72 (95% CI: 1.18–2.51) (Fig. 5). We also analyzed four studies outside Africa. The estimated prevalence in these studies was 3% (95% CI: 0–25%) (Fig. 6).

Table 2 presents the results of the subgroup analysis, employing a range of criteria including continent, sample size, NOS score, income level, diagnostic test, and publication year. The investigated prevalence rates in Africa, America, and Asia were recorded as 11%, 9%, and 1% respectively. Notably, within the African region, the prevalence of HIV demonstrated regional variation, with rates of 18% in the south, 9% in the east, and 7% in the west.

**Table 2:** The subgroup analysis results

Variables	Prevalence [95% CI]	Number of studies	Sample size	I^2^ (%)
**Diagnostic test**				
Rapid test kits	13 [10-16]	6	26,600	94%
ELISA	9 [5-16]	10	9183	96%
Self report	8 [4-16]	6	17,019	99%
**Sample size**				
≤ 1000	11 [8-14]	11	5356	93%
> 1000	9 [5-16]	11	47,446	99%
**Income**				
Upper middle income	20 [19-22]	5	8243	45%
Lower middle income	8 [5-12]	12	41,246	98%
Low income	8 [4-14]	5	3313	94%
**Years**				
Before 2015	9 [5-15]	12	38,829	99%
After 2015	11 [8-16]	10	13,973	97%
**NOS scale score**				
Low risk	10 [7-14]	15	39,952	97%
Moderate risk	10 [6-19]	7	12,850	99%
High risk	0	0	0	0
**Continent**				
Africa	11 [8-15]	18	44,946	98%
America	9 [8-11]	2	6135	57%
Asia	1 [0–62]	2	1721	94%
**Africa**				
West	7 [4-13]	2	934	82%
East	9 [6-13]	11	35,769	99%
South	18 [14-24]	5	8243	45%

In regards to diagnostic methodologies, studies implementing the rapid test exhibited a prevalence rate of 13%, while prevalence rates of 8% were observed in self-reported studies, and 9% in studies employing the enzyme-linked immunosorbent assay (ELISA) test. Distinctly, studies conducted in upper-middle-income countries displayed a prevalence rate of 20%, whereas studies conducted in lower-middle-income and low-income countries exhibited a prevalence rate of 8%.

**Fig. 7:**
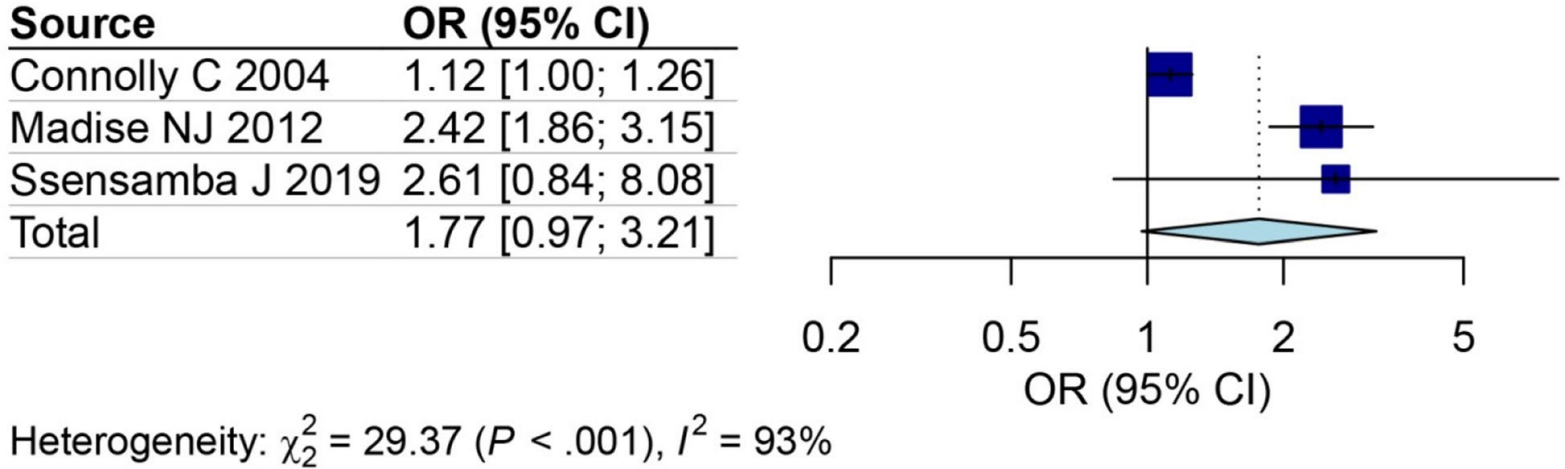
The odds ratio of HIV among individuals residing in slums area compared to non-slums area

Furthermore, the comparative analysis between slums and non-slum areas, as observed in three studies [28, 30, 41], determined the OR to assess the likelihood of HIV infection. The OR identified that individuals residing in slums area possessed a 1.77 [95% CI: 0.97; 3.21] times higher risk of contracting HIV compared to their counterparts in non-slum areas (Fig. 7).

To explore potential relationships between prevalence and key factors, meta-regression analyses were conducted based on publication year and sample size. Despite observed increases in HIV prevalence within slum areas in relation to these variables, statistical significance was not established. Specifically, the calculated *P*-values were 0.50 for sample size and 0.54 for publication year.

**Fig. 8:**
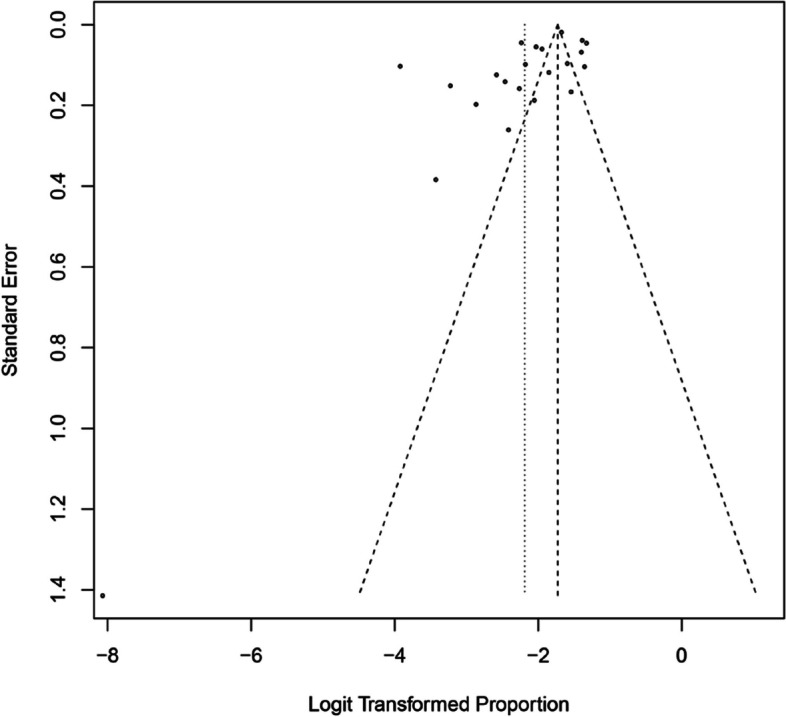
The funnel plot

The assessment of publication bias using Egger's test yielded a non-significant result, indicating the absence of bias in the studies included (*P* = 0.12). This finding is further supported by the visual representation of study distribution in the funnel plot, as depicted in Fig. 8. In the plot, the prevalence of the analyzed phenomenon is represented on the x-axis, while the standard error is plotted on the y-axis. Notably, studies with higher precision and reduced standard error tend to cluster towards the upper region of the plot, reflective of their greater accuracy. Conversely, studies exhibiting larger standard errors and clustering towards the lower end of the plot indicate lower dispersion.

## Discussion

Our study represents the systematic review and meta-analysis conducted to examine the prevalence of HIV within slum communities. A meticulous and comprehensive search was conducted, accompanied through meticulous analysis. The principal aim of this present systematic review and meta-analysis was to assess the prevalence of HIV in slums while exploring its associated factors. The overall prevalence rate of HIV infection within slums was estimated to be 10%. This observation underscores the massive burden of HIV within slum communities and emphasizes the necessity for targeted interventions that can effectively address this issue. Remarkably, slums are characterized by overcrowding, limited access to health services, poverty, and scarce resources. These conditions collectively contribute to an increased risk of HIV transmission [41]. Interventions aimed at combating HIV epidemics in slums area should focus on prevention, testing, treatment, and support services that are tailored to meet the unique needs of slum populations.

Furthermore, our analysis yielded results that align with prior research, highlighting a higher prevalence of HIV infection among women residing in slums [47, 48]. The estimated prevalence rate of HIV infection among women in slums area was found to be 13%. This gender disparity highlights important concerns and accentuates the dire need for implementing gender-specific approaches to HIV prevention and care within slum populations [49]. The OR for HIV infection among women, as compared to males, was determined to be 1.72. Consequently, it is evident that women living in slums face a significantly higher risk of HIV acquisition when compared to men [38]. This finding is consistent with the global phenomenon where women and girls disproportionally bear the burden of the HIV epidemic, particularly within resource-constrained settings and marginalized populations [48]. Addressing the gender disparity in HIV prevalence necessitates tailored interventions that acknowledge and address the unique needs and challenges faced by women residing in slum areas [50]. This includes promoting gender equality, empowering women through educational and economic opportunities, and enhancing their access to sexual and reproductive health services [51]. Providing comprehensive sexual and reproductive health services that integrate HIV prevention, family planning, and antenatal care can significantly contribute to reducing HIV transmission among slum women [52].

Numerous factors contribute to the heightened susceptibility of women residing in marginalized urban areas to contracting HIV. Gender disparities, encompassing restricted educational and economic prospects, as well as diminished agency in decision-making processes, can impede women's capacity to engage in safe sexual practices and safeguard themselves against HIV transmission. Additionally, prevailing social norms and cultural customs, exemplified by premature unions, gender-oriented violence, and transactional sexual engagements, tend to amplify the vulnerability of women to HIV contraction [37, 39, 40, 44, 45].

Our analysis uncovered a heightened probability of HIV infection among individuals residing in slums, in comparison to those living in non-slum areas. The odds ratio estimate of 1.69 implies that occupants of slum settlements endure a significantly increased vulnerability to contracting HIV. The discovery of elevated HIV infection risks within slum populations underscores the susceptibility of these communities to the HIV epidemic [53]. Slums areas are frequently characterized by overcrowding, inadequate sanitation, limited healthcare accessibility, and heightened poverty levels [32, 36]. These circumstances facilitate the transmission of HIV, including unprotected sexual activities, reduced awareness of HIV treatment and prevention methods, as well as challenges in accessing HIV testing and treatment services [54]. The amplified relative risk of HIV infection in slum areas compared to non-slum areas underscores the pressing need for targeted interventions addressing the unique obstacles encountered by slum dwellers [55]. Holistic strategies are imperative for tackling the multifaceted factors contributing to the elevated risk of HIV acquisition in slums. Interventions should concentrate on enhancing access to HIV testing and counseling services, ensuring the availability of affordable and high-quality healthcare services, promoting HIV prevention education, and facilitating the linkage to HIV care and treatment for individuals who test positive [39, 41, 43]. Furthermore, it is essential to address the social determinants of health, such as poverty, unemployment, and inadequate housing, in order to diminish the vulnerability of slum populations to HIV infection [56]. Community-based approaches with active participation from community members, local leaders, and organizations have demonstrated remarkable efficacy in addressing the specific challenges faced by slum populations [57]. To effectively tackle these challenges, these approaches should encompass targeted outreach, peer education, and the establishment of support networks, all aimed at enhancing awareness, promoting safe sexual practices, and encouraging regular HIV testing [47, 54]. It is crucial to foster collaboration amongst healthcare providers, community organizations, community members, and policymakers to develop and implement interventions that yield positive outcomes [55]. Moreover, to ensure sustainable and equitable access to healthcare services and social support systems in slums, policy changes and resource allocation are imperative [58]. It is important to note that variations in HIV prevalence estimates, resulting from different testing methods, serve as a key factor to consider when interpreting the findings of this study [59]. Our analysis has revealed disparities in prevalence estimates based on the employed testing method, encompassing rapid tests, self-reports, and ELISA techniques.

These variations underscore the necessity of implementing standardized testing protocols in research and surveillance endeavors pertaining to HIV prevalence. The objective of standardization is to ensure the systematic and trustworthy gathering of data, thereby facilitating accurate and comparable prevalence estimations across diverse studies and populations [60]. In resource-limited settings, rapid tests are frequently employed due to their straightforward nature, rapid turnaround times, and user-friendliness [61]. Nonetheless, these rapid tests may exhibit shortcomings in terms of sensitivity and specificity when compared to more sophisticated laboratory-based assays, such as the ELISA [26]. Consequently, prevalence estimations based on rapid tests may deviate from those obtained using more sensitive and specific methodologies. Another methodology utilized in certain studies for prevalence estimation is self-reporting of HIV status [31]. However, self-reporting is susceptible to potential biases, including under-reporting due to social stigma or over-reporting due to recall bias [62]. Consequently, significant disparities can arise between self-reported prevalence figures and estimates derived from alternative testing modalities. ELISA, an extensively embraced and validated laboratory-based assay, is renowned for its exceptional sensitivity and specificity. Nevertheless, its utilization necessitates specialized equipment and skilled personnel, thereby rendering it less viable within resource-limited settings. Nonetheless, ELISA furnishes a more precise and reliable appraisal of HIV prevalence compared to rapid tests or self-reporting [34]. The divergent prevalence estimations stemming from different testing methodologies serve as a conspicuous reminder regarding the paramount importance of employing standardized protocols to ensure the accuracy and comparability of data across diverse studies [33, 45]. These standardized testing protocols should encompass meticulous quality control measures, compliance with established testing algorithms, and appropriate validation of the testing methodologies [56]. Moreover, it is imperative to recognize the limitations and potential biases associated with each testing modality when interpreting prevalence estimations [36, 41, 42]. Comprehensive approach that incorporates multiple testing methods, when feasible, can provide a more robust understanding of the HIV prevalence in slums.

Our analysis has revealed significant regional disparities in the prevalence of HIV, thereby shedding light on the differential burden of HIV across distinct geographical settings. The findings elucidate that within slum areas, Africa manifests the highest prevalence of HIV at 11%. Contrarily, the Americas display a prevalence rate of 9%, while Asia exhibits the lowest prevalence at 1%. These inter-regional discrepancies in HIV prevalence are indicative of the heterogeneous epidemiological contexts and dynamics of the HIV epidemic. Historically, Africa has borne the brunt of the HIV epidemic, characterized by high prevalence rates in numerous countries [49, 51]. This can be attributed to a multitude of factors, including but not limited to, multiple sexual partnerships, limited accessibility to prevention and treatment services, cultural norms, and socio-economic challenges [48]. Conversely, the lower prevalence observed in the Americas and Asia may be attributed to variations in the distribution of risk factors, concerted preventive efforts, improved access to healthcare, and differing cultural and social practices [45]. Notably, the implementation of comprehensive HIV prevention and treatment programs within the Americas has resulted in decreased HIV transmission rates and enhanced access to care [38, 39]. Similarly, in Asia, the relatively low overall HIV prevalence can be attributed to the efficacy of prevention strategies, robust public health interventions, and targeted initiatives focused on key populations [46]. These regional discrepancies in HIV prevalence underscore the imperative of context-specific interventions tailored to the unique challenges and dynamics exhibited by each respective region. Such interventions should duly consider the social, cultural, economic, and structural factors that contribute to HIV transmission within specific geographic areas [40]. In regions characterized by high HIV prevalence, such as Africa, interventions should place paramount emphasis on scaling up prevention strategies, expanding access to HIV testing and counseling services, and ensuring the availability and accessibility of antiretroviral treatment for individuals living with HIV [42]. Comprehensive sexual and reproductive health services, community-based awareness campaigns, and harm reduction programs have been identified as pivotal interventions aimed at reducing new infections within slum communities [39]. In slums area, it is imperative to not only sustain but also strengthen prevention efforts, ensuring early diagnosis and improving access to care [19, 39]. Additionally, the implementation of ongoing surveillance, effective health education, and targeted interventions for key populations at higher risk, particularly within slum areas, is crucial in maintaining a low prevalence of infections and preventing future escalations [46]. However, it should be noted that the number of studies conducted in continents other than Africa is comparatively limited, thus limiting definitive conclusions to be drawn from a phenomenon solely based on the findings of two studies with relatively small participant sizes.

### Limitations

Our study encountered a number of limitations that warrant acknowledgement in an academic context. First, it is important to note the significant heterogeneity observed among the selected studies. This heterogeneity can be attributed to various factors, including discrepancies in study populations, differences in sampling methods, variations in diagnostic techniques, and variances in socioeconomic and cultural contexts. As a result, it is crucial to approach the interpretation of the pooled prevalence estimates with caution, acknowledging the presence of such heterogeneity. Despite the aforementioned limitations, the overall estimate still provides valuable insights into the general magnitude of HIV prevalence in slums area. To account for and explore the heterogeneity observed, our study employed regression and subgroup analyses. Additionally, we utilized the Baujat plot and meta-regression techniques to investigate factors influencing the high level of heterogeneity. However, it is important to acknowledge that these approaches have their own limitations. Another notable limitation is the paucity of prevalence studies in many countries. Conducting such studies in different countries, particularly in impoverished and suburban areas, would greatly contribute to planning and decision-making processes for service provision in these regions. Furthermore, it is worth mentioning that several of the included studies failed to investigate potential risk factors specific to slum areas. Understanding these risk factors is key to gaining insights into the underlying causes of the high prevalence among slum residents. It is essential to acknowledge that our study's inclusion criteria focused solely on English-language texts in systematic reviews and meta-analyses. This selection approach stems from practical considerations such as resource constraints and language barriers. However, this practice can introduce biases and limitations. The exclusion of non-English studies may reduce the comprehensiveness of the review. The prominence of the English language in the academic sphere, along with the easier access to English studies due to institutional subscriptions, contribute to the preferential inclusion of English-language texts in our study.

The generalizability of the findings should be interpreted in the context of certain limitations and contextual factors. This systematic review and meta-analysis present valuable insights into the prevalence of HIV in slum areas; however, it is important to acknowledge the regional variations that have been observed. The higher prevalence of HIV in Africa compared to the Americas and Asia emphasizes the influence of distinct epidemiological dynamics, risk factors, and healthcare infrastructures. Additionally, the variations in prevalence estimates, which are dependent on different testing methods, necessitate cautious interpretation. These findings offer valuable information that is specific to the context of the regions with similar settings and challenges. However, their applicability to other populations requires careful consideration of local conditions.

The implications of our study for public health decision-making are significant, as they necessitate targeted interventions to address the heightened risk of HIV in slum areas. Comprehensive strategies are needed to address factors such as poverty, limited healthcare access, and gender disparities, which would guide the implementation of effective prevention and support programs. While it is necessary to exercise caution in directly generalizing our findings due to regional variations, they serve as a foundation for evidence-based decision-making aimed at reducing the burden of HIV and enhancing well-being in similar contexts. This can be achieved through collaboration among healthcare professionals, communities, and policymakers.

## Conclusion

The prevailing prevalence of HIV infection within slum communities carries profound ramifications for public health. This underscores the pressing imperative to enact focused interventions aimed at mitigating HIV transmission and enhancing the healthcare outcomes of those living in slums. These interventions encompass a broad spectrum of measures, including comprehensive sexual and reproductive health services, HIV testing and counseling, dispensation of condoms, facilitated access to antiretroviral therapy, harm reduction initiatives targeting substance abuse, and community-based endeavors to raise awareness and impart education.

### Supplementary Information

**Additional file 1:** **Appendix 1. **PRISMA checklist.

**Additional file 2:** **Appendix 2. **The search strategy.

**Additional file 3:** **Appendix 3. **The risk bias assessment using NOS.

**Additional file 4:** **Appendix 4. **Sensitivity analysis.

## Data Availability

The data supporting the findings can be found in the main paper.

## Abbreviations

HIV: Human immunodeficiency virus
PrEP: Pre-exposure prophylaxis
ART: Antiretroviral therapy
PRISMA: Preferred reporting items for systematic review and meta-analyses
DOAJ: Directory of open access journals directory
NOS: Newcastle–Ottawa scale
CI: Confidence intervals
RR: Relative risk
OR: Odds ratio
ELISA: Enzyme-linked immunosorbent assay
